# Retraction Note: Acupuncture elicits neuroprotective effect by inhibiting NAPDH oxidase-mediated reactive oxygen species production in cerebral ischaemia

**DOI:** 10.1038/s41598-022-09103-1

**Published:** 2022-03-23

**Authors:** Guang-Xia Shi, Xue-Rui Wang, Chao-Qun Yan, Tian He, Jing-Wen Yang, Xiang-Hong Zeng, Qian Xu, Wen Zhu, Si-Qi Du, Cun-Zhi Liu

**Affiliations:** Acupuncture and Moxibustion Department, Beijing Hospital of Traditional Chinese Medicine Affiliated to Capital Medical University, 23 Meishuguanhou Street, Dongcheng District, Beijing, 100010 China

Retraction of: *Scientific Reports* 10.1038/srep17981, published online 10 December 2015

The Editors have retracted this Article.

After publication of this Article concerns have been raised about the overlap of Figure 1 with another article from the authors, which was not cited in the Article^1^. Subsequent investigation showed that overlap extends also to the data shown in Figure 2. Data shown in this figure is presented as coming from a different sample in the other article. Due to the time since the research was performed, the Authors are not able to provide the original data underlying this study. Editors therefore no longer have confidence in the conclusions of this Article.

Guang-Xia Shi, Chao-Qun Yan, Tian He, Jing-Wen Yang, Qian Xu and Cun-Zhi Liu disagree with the retraction. Xue-Rui Wang, Xiang-Hong Zeng, Wen Zhu and Si-Qi Du did not respond to the correspondence from the Editors.
